# A Quality Improvement Initiative to Control a Gram-negative Sepsis Outbreak in a Resource-limited Neonatal Intensive Care Unit Using Systems Engineering Initiative for Patient Safety and a Modified Donabedian Framework

**DOI:** 10.1097/pq9.0000000000000869

**Published:** 2026-02-23

**Authors:** Kedar Sawleshwarkar, Mahtab Singh, Ramesh Bajaj, Saumya Sawleshwarkar, Chintamani Dande

**Affiliations:** From the * Department of Neonatology, Deogiri Children’s Hospital, Chhatrapati Sambhajinagar, Maharashtra, India; † Department Of Pediatrics, R K Damani Medical College, Chhatrapati Sambhajinagar, Maharashtra, India; ‡ Niwara Healthcare Improvement LLP India, Dwarka, New Delhi, India.

## Abstract

**Introduction:**

Each year, 2.6 million newborns die globally, making up 46% of mortality in children younger than 5 years. Most of these deaths (96%) occur in low- and middle-income countries, with India accounting for approximately 25% of global neonatal mortality. Healthcare-associated infections (HCAIs) are a major cause of morbidity and mortality in neonatal intensive care units across low- and middle-income countries, with incidence rates ranging from 15.2 to 62.0 per 1,000 patient-days. A simplified and scalable framework is required to reduce neonatal mortality from sepsis.

**Methods:**

We experienced a Gram-negative sepsis outbreak in our neonatal intensive care unit beginning in July 2021, with HCAI peaks reaching 30–40 per 1,000 patient-days and a case fatality of approximately 30%. The outbreak was contained through a strategic combination of the Modified Donabedian approach and the application of the Systems Engineering Initiative for Patient Safety model.

**Results:**

HCAIs decreased from a mean of 14 and a peak of 38.7 per 1,000 patient-days to a sustained mean of 2.9, and antibiotic use decreased by 50%. Late-onset sepsis–related mortality fell from 27.3% in 2021 and 40% in 2022 to 12% in 2023 and 0% in 2024.

**Conclusions:**

Effective outbreak control requires a holistic, structured approach that encompasses all components of the Systems Engineering Initiative for Patient Safety model, namely studying the work system (structure), processes, and outcomes. Activating agency and fostering psychological safety transform healthcare workers from being recipients of policy into active participants in implementing solutions. Strong leadership galvanizes teams toward a shared purpose and overcomes significant challenges to improve care.

## INTRODUCTION

Each year, 2.6 million newborns die globally, making up 46% of mortality in children younger than 5 years. Ninety-six percent of the deaths occur in low- and middle-income countries (LMIC), with India accounting for approximately 25% of global neonatal mortality.^1,2^ Healthcare-associated infections (HCAIs) are a major cause of morbidity and mortality in neonatal intensive care units (NICUs) across LMIC countries, with the HCAI incidence in the NICUs estimated at 15.2–62.0 per 1,000 patient-days, up to 9 times higher than that observed in some high-income settings. HCAIs are associated with increased costs and longer lengths of stay.^2^

Almost 80% of the total neonatal deaths in the world (2.4 million) occur in sub-Saharan Africa and Southern Asia. The Sustainable Development Goals set by the United Nations include a target to reduce the neonatal mortality rate to less than 12 per 1,000 live births by 2030. India currently has a neonatal mortality rate of around 20, with a large intrastate variation.^2^ A simplified and scalable framework is required to improve outcomes in neonatal sepsis and reduce neonatal mortality.

### Literature Review

Reports of outbreaks caused by Gram-negative bacteria predominate, with 21 of 39 (54%) outbreaks occurring in NICUs.^1^ A meta-analysis revealed that 31% of infected neonates died in these outbreaks. Fifty-seven percent of the 75 included studies failed to identify a source of the outbreak, whereas 15% identified an index case with subsequent horizontal transmission.^1^

The German Commission for Hospital Hygiene and Infectious Disease Prevention (KRINKO) recommended extending active surveillance cultures to all neonates admitted to the NICU to detect colonization clusters.^3^ Active surveillance programs are rare in low-resource settings.

Most published studies describe a multifaceted approach to infection prevention and control in response to an outbreak. The interventions included staff and parent education on hand hygiene, patient isolation, additional contact precautions, an increase in the staff-to-patient ratio, and enhanced environmental cleaning with increased disinfectant concentration and screening.^1–3^ Removal of a specific contaminated product, such as ultrasound gels, has been advised.^4^ Contaminated unpasteurized donor breast milk ultimately resulted in the cessation of the shared milk program in Japan.^5^ In rare cases, prolonged or severe outbreaks led to temporary or permanent ward closures.^6^

Brooks et al^7^ demonstrated the impact of the NICU environment on the colonization of neonates from surfaces to the gut. Healthcare workers’ hands have been shown to harbor variable amounts of potential fecal colonizers.^7^ The same bacterial species were concurrently isolated from parents’ hands and their mobile phones.^8^

### Problem Statement

This improvement report is from a standalone 20-bed level 3 NICU in a resource-limited tier 2 city of Chhatrapati Sambhajinagar, Maharashtra, India. This facility is also a teaching institute with a fellowship program in neonatology. Recommended bed space and nurse–patient ratios are 2 limiting factors in our unit. We experienced a Gram-negative sepsis outbreak in our NICU in 2021. During a period of 15 months, 23 very low birth weight (<1,500 g) babies developed late-onset sepsis (LOS). A cluster of sepsis cases occurred around October 2021. Sixteen cases were culture-positive for sepsis and grew Gram-negative organisms, including *Klebsiella*, *Enterobacter*, *Escherichia coli*, and *Pseudomonas*. The outbreak mainly involved extreme preterm neonates (<28-wk gestation). Seven babies out of 23 died due to either sepsis or late complications associated with sepsis, such as necrotizing enterocolitis. The mean gestational age was 29.3 weeks, and the mean birth weight was 1,160 g among the babies affected by sepsis.

## METHODS

### Ethics Approval

Ethical committee approval was not required, as the project was conducted as a quality improvement initiative.

### Reporting

Standardsfor Quality Improvement Reporting Excellence 2.0 guidelines guided the preparation of this article. Data were extracted from the inpatient electronic health records into Excel spreadsheets. The interventions were planned and implemented using the Systems Engineering Initiative for Patient Safety (SEIPS) framework.

### Operational Definitions

LOS: Defined as sepsis acquired in the NICU after 6 days of admission or after 3 days in those babies in whom there were no risk factors for early-onset sepsis, such as preterm premature rupture of membranes, maternal fever, or evidence of infection in the mother, and whose admission blood culture was sterile.^9^

Clinical sepsis: Defined as a positive sepsis screen and clinical suspicion warranting broad-spectrum antibiotic therapy for a period of 5 or more days despite a negative blood culture.

Sepsis outbreak: Defined as 2 or more linked episodes of sepsis with the same or similar infectious agent occurring in the same healthcare setting during a specified period of time (<2 wk) or a higher-than-expected number of nosocomial sepsis cases in a given healthcare area during a specified period of time.

Control of outbreak: Defined as the absence of more than 1 culture-positive Gram-negative bacterial LOS within a 4-week period and sustaining it for at least 3 months.

### Interventions

**Fig. 1.**
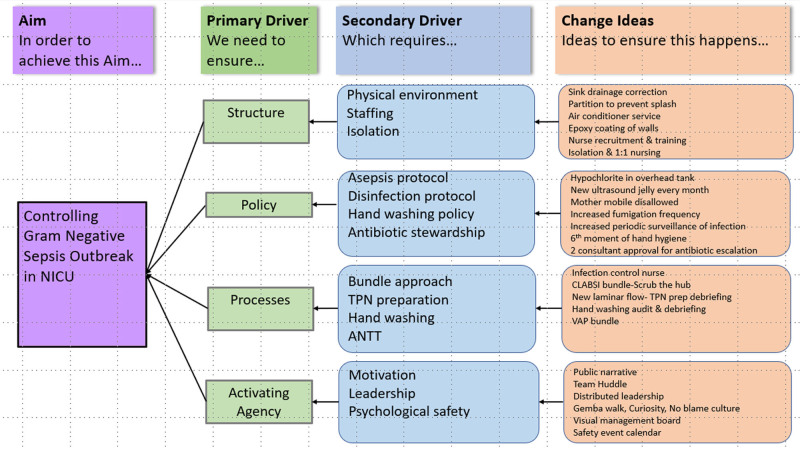
Driver diagram: theory of change for control of sepsis. ANTT indicates Aseptic non-touch technique; CLABSI, Central line associated blood-stream infection; VAP, Ventilator- associated pneumonia.

We developed a key driver diagram (Fig. 1) to describe our theory of change for controlling sepsis.

The SEIPS model provides a structured approach to healthcare quality, emphasizing the dynamic interplay among the work system, processes, and outcomes. The structure of an organization (work system) affects how safe care is provided (the process); and the means of caring for and managing the patient (the process) affect the patient outcome. Although planning our interventions, we used the Donabedian framework of structure, process, and outcomes, incorporating culture. We validated our results using the SEIPS model to demonstrate the effectiveness of our interventions, ranging from initial structural changes to profound behavioral shifts.^10,11^

#### Process Indicators

Rapid, overlapping Plan-Do-Study-Act cycles were conducted, as documented below, for interventions such as handwashing audits, scrub the hub for central lines, total parenteral nutrition (TPN) preparation, and other structural and policy changes. Therefore, no separate process indicators could be tracked, although debriefing and rapid adjustments were undertaken, considering the urgency to contain the outbreak.

#### Outcome Indicators

The following outcome indicators were tracked:

1. HCAIs incidence: number of HCAIs per 1,000 patient-days per month.
2. Broad-spectrum antibiotic prescription: percentage of admitted newborns receiving broad-spectrum antibiotics (piperacillin, meropenem, vancomycin, colistin, etc.) for sepsis per month.
3. Percentage of deaths attributable to LOS per year.

#### Balancing Indicators

A verbal survey conducted among staff expressed high work overload and high stress levels during the outbreak, which were relieved to some extent after the outbreak was contained and were much lower after a sustained reduction in HCAIs. A formal measurement of these balancing indicators was not undertaken.

#### Interventions

The interventions assessed each element of the work system as defined by the SEIPS model.

##### People (Patients)

A multidisciplinary team including consultants, residents, a microbiologist, nursing staff, and maintenance personnel convened for root cause analysis, intending to control the outbreak as early as possible. Discussions of the outbreak yielded critical information:

1. Most babies were extremely low birth weight (<1,000 g), and all were less than 1,500 g.
2. The babies who developed sepsis were located in a single area close to the handwashing sink and the air conditioner outlet.
3. Almost all babies were receiving some form of respiratory support, either invasive or noninvasive, from the time of admission.
4. All babies were receiving TPN via an umbilical venous catheter.
5. Babies were relatively stable until day 6 or 7, after which they became symptomatic and developed sepsis.

#### Environment

Initially, we assessed potential environmental contaminants and implemented measures to enhance infection control in the NICU (Table 1). A brief lull in sepsis cases was observed; however, a recurrence was subsequently noted.

**Table 1.** SEIPS Work System Interventions (Structure) and Early Process Changes

Category	Intervention	Description
Initial work system interventions (structure)		Focused on immediate physical and policy-based changes
Environmental adjustments	Hand washing sink	Drainage corrected; frequency of disinfection increased
	Barrier installation	Glass–aluminum barriers installed to prevent splash contamination
	Air conditioner maintenance	Monthly disinfection by trained technicians
Evolving work system and policy changes		Recognizing limitations in altering fixed structural elements, our focus shifted to rapid, actionable changes within the environment and initial process adjustments
Environment: Structural refinement	NICU walls repair	Epoxy coating applied to NICU walls to eliminate microbial crevices
Environment: Isolation	Neonatal cohorting	Septic neonates isolated in a dedicated bay with PPE-equipped staff
Task: Disinfection	Fumigation	Regular fumigation of NICU bays during windows of opportunity
Task: Workforce strengthening	Nurse–patient ratio	Additional nurses recruited and trained in infection control
Environment: Water safety	Water disinfection	Hypochlorite is added to the overhead tank for full system disinfection

PPE, Personal Protective Equipment.

#### Tools and Technology

We assessed the required tools for preventing infections.

#### Tasks

We ensured that the clinical staff received training in infection control.

Despite these initial interventions, the sepsis outbreak persisted, underscoring the need for profound systemic changes (Table 2).

**Table 2.** SEIPS Deep Dive Into Process and Policy Changes

Category	Intervention	Description
Tool: Hand hygiene	Hand scrub reform	Replaced povidone iodine with chlorhexidine 2.5% + 70% alcohol
Task: Audit	Hand hygiene monitoring	World Health Organization tool audits, video debriefs, and observation were introduced to improve the steps of handwashing
	Sixth moment	Handwashing is mandated before NICU entry
Task: Feed preparation and medication	Formula water preparation	New stainless-steel kettle procured; disinfection training conducted
Tool: Medication	Probiotic introduction	Bifidobacterium breve is given to all very low birth weight neonates to prevent necrotizing enterocolitis
Tool: Antibiotic stewardship	Dual consultant approval	Required for escalation to higher antibiotics
Task: CLABSI prevention	Scrub the hub	Reinforced daily scrub-the-hub practice for central lines
Task: Equipment and asepsis	Laminar flow use	Hood replaced; video-recorded TPN preparation; Standard Operating Procedure developed; 2-person technique implemented
Task: Disinfection	Warmer disinfection	Shifted to scrubbing to eliminate biofilms on radiant warmers
Task: Oversight	Infection control nurse	Senior staff designated to oversee disinfection processes

CLABSI, Central line associated blood-stream infection.

Further interventions targeted high-risk procedures and environments, underscoring our commitment to systemic improvement.

#### Organization

##### Organizational Culture: Activating Agency

Despite emotional fatigue and frustration, we realized that structural and process changes alone were insufficient (Table 3). Activating collective agency—through behavioral and cultural interventions—to drive ownership and sustain change was critical for improvement and for maintaining the gains achieved.

**Table 3.** SEIPS Organizational Culture Interventions: Activating Agency

Category	Intervention	Description
Psychological safety	Feedback culture	Nonblaming observations and feedback during debriefs
Distributed leadership	Peer observation	Empowered staff through mutual monitoring and accountability
	Infection control deputation	An infection control nurse appointed from the internal staff
Adaptive learning	Continuous reflection	Real-time debriefs, process adjustments based on feedback
Public narrative	Story of self/us/now	Narrative used to align the team through shared experiences and purpose
Organizational alignment	Vision, mission, values	Integrated into daily huddles to reinforce purpose and unity

## RESULTS (OUTCOME)

**Fig. 2.**
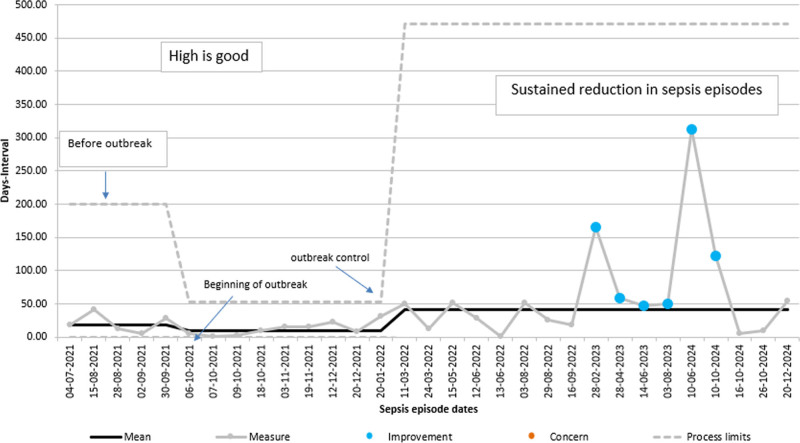
t-Chart for Sepsis events: days between sepsis episodes.

The t-chart for sepsis events (Fig. 2) tracks the number of days between consecutive sepsis episodes in the NICU. Between June and August 2021, the system shows moderately stable performance, with intervals ranging from 18 to 42 days between events.

### Outbreak Phase (Mid-2021)

The chart shows a progressive decline in the interval between sepsis episodes from September 2021 onward (5–16 d) during the outbreak. Multiple consecutive points from October to December 2021, clustered below the mean (interval 3–16 d), reflect uncontrolled infection spread and frequent sepsis episodes, confirming process instability.

### Outbreak Control Phase (2022)

Following interventions, the intervals between sepsis events began to increase (from February 2022 onward, 31–52 d). By mid-2022, the system displays an increasing number of intervals above the mean, signaling improvement in infection control practices.

### Sustained Improvement (March 2023 Onward)

After the outbreak control, the t-chart shows prolonged intervals, including outliers at 165, 312, and 122 days. A shift in the mean from 9.42 to 40.7 is observed. This change indicates a new stable system with substantially fewer sepsis events over time. The upper process limit also shifts upward, reflecting a new, improved process capability. Through 2023–2024, the system maintains extended intervals between events (47–122 d), indicating ongoing control and resilience.

### Process Stability

Although occasional shorter intervals reappeared, the overall trend shows a sustained upward shift, confirming special-cause improvement. This stability corresponds to a reduction in HCAIs, as demonstrated in the HCAI XmR-chart.

### XmR-chart: Statistical Process Control HCAI Incidence

#### Baseline Phase (January 2021–June 2021)

**Fig. 3.**
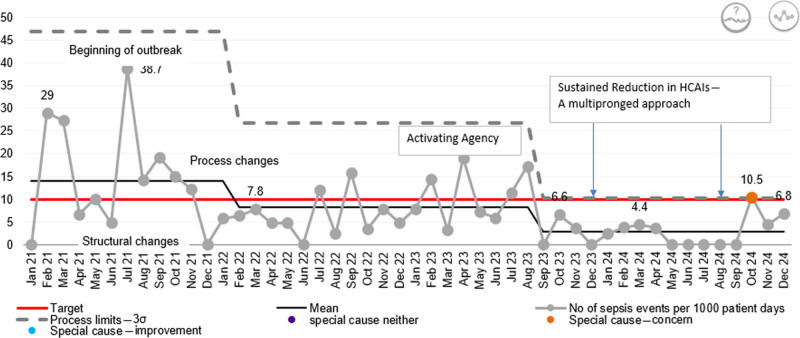
XmR-chart: incidence of HCAIs per month per 1,000 patient-days.

HCAI rates demonstrated high variability from low single digits to a spike of 29 per1,000 patient-days in February 2021 (Fig. 3).

#### Outbreak Phase (2021)

A marked elevation in HCAI rates was observed, peaking at 38.7 of 1,000 patient-days (June 2021) at the onset of the outbreak. Multiple points exceeded the mean HCAI of 14, indicating instability in the infection control process.

#### Early Interventions (Structural and Process Changes, Late 2021 to Early 2022)

Targeted measures, including environmental modifications, resulted in a progressive decline in infection rates.

#### Outbreak Control (February 2022 Onward)

The chart shows a sustained downward shift in HCAIs, confirmed by a cluster of consecutive points below the previous centerline resulting in a new, lower statistical mean (8.23).

#### Behavioral Reinforcement Phase (Mid-2022–2024)

Sustained improvement was achieved through reinforcement of staff behaviors and compliance with established protocols, with only occasional points approaching upper control limits, suggesting continued stability.

#### Overall Signal

The XmR-chart demonstrates special-cause variation leading to sustained improvement, attributable to a multipronged approach (structural changes, process redesign, and behavioral interventions). The control limits narrowed over time with a shift in the mean (2.90), reflecting a reduction in variability and an increase in the reliability of infection prevention practices.

**Fig. 4.**
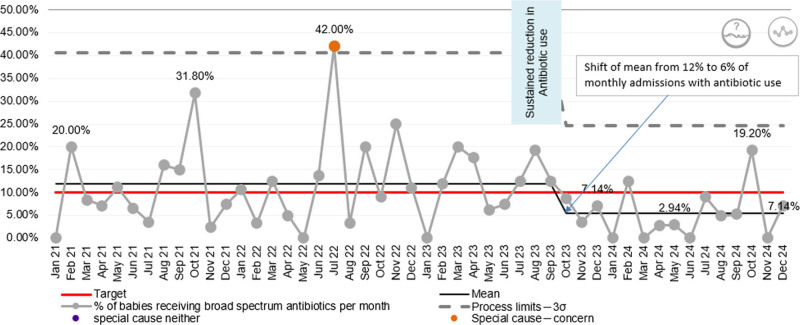
P-chart: percent of NICU admissions receiving broad-spectrum antibiotics.

The P-chart (Fig. 4) for the percent of NICU admissions receiving broad-spectrum antibiotics per month shows considerable month-to-month variation around a mean of approximately 12% with peaks of 31.8% in October 2021 and 42% in July 2022, reflecting a mix of episodic clinical concerns and a reactive approach to the outbreak.

#### Postintervention Phase

Targeted infection control measures resulted in a marked downward shift in antibiotic use beginning in September 2023, with the mean dropping to 6%, a 50% reduction in antibiotic use, indicating a true process change rather than random fluctuation, except for an isolated peak of 19.2% in October 2024. Therefore, the Statistical Process Control not only reflects improved antibiotic stewardship but also indirectly validates the effectiveness of infection control interventions.

The percentage of deaths related to LOS among total NICU deaths showed a progressive decline from 27.3% in 2021 to 40% in 2022, then to 12% in 2023, and to 0.0% in 2024.

## DISCUSSION

Jain et al,^12^ in their study of 5 special newborn care units in district hospitals in India, reported a case fatality of culture-positive sepsis of 36.6%, which is similar to 30.4% mortality in our report. Mirek Skrypak^13^ mentioned an approach to improving care quality with 4 components based on the Donabedian model of systems (structure + process + culture = outcomes). This approach aligns with the SEIPS model.

We effectively used a similar approach, addressing both structural and process elements, as well as behaviors, using the Institute for Healthcare Improvement Psychology of Change framework.^14^ We labeled it as a Modified Donabedian approach to improve the quality of care.^13,14^

Our experience offers several key lessons that are applicable to low-resource settings.

Yu et al^15^ reported that an outbreak of *Enterobacter cloacae* was likely caused by multiple modes of transmission, including a contaminated saline bottle as an initial common source. They concluded that effective infection control requires a multidisciplinary approach and reinforcement of infection control procedures, including aseptic technique, handwashing, proper isolation, and disinfection of environmental surfaces. We also implemented numerous structural and process changes, including reinforcing infection control measures as described by Yu et al.^15^ Gastmeier et al^16^ concluded that in 48.6% of NICU outbreaks, they were unable to identify the sources. We believed that the outbreak could result from failure in multiple processes, as well as contextual structural elements such as bed spacing, nurse–patient ratios, and environmental factors that contributed to the spread of infection.

A thorough analysis of environmental factors is essential. The Neonatal Care in Scotland: A Quality Framework^17^ recommends a 1:4 staff-to-baby ratio in a special care unit, a 1:2 staff-to-baby ratio in a high dependency unit, and a 1:1 staff-to-baby ratio in intensive care. The recommended space allowance for an open bay in the intensive care and high-dependency units is 13.5 square meters (sqm) for the clinical area, increasing to 20 sqm per single room when access space and shared space for core support (eg, pharmacy and storage) are considered. In special care units, the recommended space allowance is 9 sqm, rising to 11.5 sqm when access space and shared space for core support (such as pharmacy and storage) are included. Admissions of many high-acuity infants in a short period strain the staff-to-baby ratio in our unit.

Additional considerations include the following:

- Accommodation requirements, which are another contextual limitation in settings like ours in the LMIC.^17^
- Specific source of infection, such as contaminated parenteral nutrition, could not be identified,^17^ and therefore, we reviewed and improved many processes of care, including parenteral nutrition, as described earlier in interventions.
- Clinical effectiveness of microbiological surveillance of staff, care equipment, and the environment screening for Gram-negative bacteria other than *Pseudomonas* is unproven.^17^
- Our periodic staff and environmental surveillance were inconclusive.

### Sustainability Measures

To sustain these improvements, we have outlined the following strategic pillars:

1. Sustained vigilance and regular auditing of all infection control protocols.
  - A Standard Operating Procedure for TPN preparation and a comprehensive disinfection protocol for the NICU were developed under the leadership of a nurse who was later appointed as the infection control nurse.
  - Daily morning team huddles incorporated discussions on safety and quality measures, ensuring ongoing vigilance and team alignment.
  - Hand hygiene compliance is regularly audited using the World Health Organization tool.
2. Continuous training for all staff to align with evolving best practices.
3. Feedback loops for staff input and iterative protocol adaptation.
  - Following each sepsis event, a root cause analysis is performed, followed by the implementation of appropriate corrective measures.
  - To enhance transparency and shared learning, safety and quality events are tracked on a calendar, and sepsis incidence, along with higher antibiotic usage, are plotted on Statistical Process Control charts. These charts are prominently displayed in the NICU to promote awareness and accountability among all team members.
4. Resources required for effective disinfection are ensured, and the designated staff maintains inventory.
5. Leadership is key for ongoing success.
  - The successful containment of the sepsis outbreak is viewed not as a conclusion, but as a significant milestone in our ongoing journey toward excellence in patient safety and quality care.
  - Leadership engagement is reinforced through Gemba walks and intentional, frequent interactions between leaders and frontline staff, aimed at fostering motivation and alignment with the shared purpose of patient safety.

## LIMITATIONS

The interventions were introduced in an overlapping, iterative manner rather than sequentially or simultaneously, so we could not measure their individual impact. However, we are of the firm opinion that such an outbreak of infection requires a multipronged approach to control, as noted by Johnson and Quach^1^ in their review of the literature on outbreaks in the NICU.

Due to contextual limitations, we were unable to address structural elements such as bed spacing and area per bed. We did not use advanced molecular investigations, such as whole-genome sequencing, due to economic constraints. Monthly variations in the number of admissions and the acuity of care, as reflected by gestational age, birth weight, and the need for clinical support, can influence outcome measurements. However, the effect of this limitation is likely minimized given the long data collection period. We did not calculate the cost of care, so a comparison is not available to understand the effect of infections on the cost of care and length of stay.

## LEARNING AND CONCLUSIONS

The analysis identified 4 key interventions for targeted improvement:

1. Effective outbreak control requires a holistic and structured approach encompassing all components of the SEIPS model, namely the work system (structure), processes, and outcomes.
2. Data-driven iterative change with continuous surveillance, audit, and debriefing is essential for identifying gaps and refining interventions.
3. Empowering staff, activating the agency, and fostering psychological safety transform staff from recipients of policy into active participants in solutions.
4. Strong leadership, even within low morale, can galvanize a team toward a shared purpose and overcome significant challenges.

This integrated approach, driven by a commitment to quality and safety, was pivotal in regaining control of the outbreak and sustaining reductions in HCAIs and antibiotic use. This approach is scalable to similar healthcare settings across the globe, especially in LMIC.

## ACKNOWLEDGMENTS

The authors acknowledge the consultants, fellows, and nursing and administrative staff of Deogiri Children’s Hospital for their support.
